# Electroacupuncture produces analgesic effects via cannabinoid CB1 receptor-mediated GABAergic neuronal inhibition in the rostral ventromedial medulla

**DOI:** 10.1186/s13020-025-01083-4

**Published:** 2025-03-04

**Authors:** Kexing Wan, Qian Xu, Yulong Shi, Chi Cui, Jie Lei, Kailing Zhang, Qingxu Yao, Yiqing Rao, Ziyu Zhou, Yisong Wu, Jiale Mei, Hui-Lin Pan, Xianghong Jing, He Zhu, Man Li

**Affiliations:** 1 School of Basic Medicine, Tongji Medical College, Hubei key Laboratory of Drug Target Research and Pharmacodynamic Evaluation, Huazhong University of Science and Technology, Wuhan, 430030 China; 2 Institute of Acupuncture and Moxibustion, China Academy of Chinese Medical Sciences, Beijing, 100700 China; 3 Department of Anesthesiology and Perioperative Medicine, The University of Texas MD Anderson Cancer Center, Houston, TX 77030 USA; 4 Department of Clinical Research Institute, Central People’s Hospital of Zhanjiang, Zhanjiang, 524000 China; 5 Clinical College of Chinese Medicine, Hubei University of Chinese Medicine, Wuhan, 430061 China

**Keywords:** Acupuncture, Inflammatory pain, Neuropathic pain, Cannabinoid Receptors, Rostroventromedial Medulla, GABA neuron

## Abstract

**Objective:**

Electroacupuncture (EA) is commonly used for pain control in clinical practice, yet the precise mechanisms underlying its action are not fully understood. The rostral ventromedial medulla (RVM) plays a crucial role in the modulation of pain. GABAergic neurons in the RVM (GABA^RVM^ neurons) facilitate nociceptive transmission by inhibiting off-cells activity. This research examined the role of GABA^RVM^ neurons in the analgesic effects of EA.

**Methods:**

Nociceptive behavior was evaluated using inflammatory pain models induced by complete Freund's adjuvant (CFA) and neuropathic pain models induced by chronic constrictive injury (CCI). Also, in situ hybridization, chemogenetics, in vivo mouse calcium imaging, and in vivo electrophysiological recordings were used to determine neuronal activity and neural circuitry.

**Results:**

EA at the “Zusanli” (ST36) on the affected side produced a significant analgesic effect in both CFA and CCI models. CFA treatment and CCI elevated the calcium activity of GABA^RVM^ neurons. Also, EA reduced the calcium activity, neuronal firing rates, and c-Fos expression of GABA^RVM^ neurons in both pain models. Chemogenetic inhibition of GABA^RVM^ neurons increased nociceptive thresholds. Chemogenetic activation of GABA^RVM^ neurons caused increased pain sensitivity in control mice and negated the analgesic effects of EA in both pain models. Moreover, reducing cannabinoid CB1 receptors on GABA^RVM^ neurons counteracted the analgesic effects of EA in CFA and CCI-induced pain models.

**Conclusions:**

The study indicates that the analgesic effect of EA in inflammatory and neuropathic pain is facilitated by CB1 receptor-mediated inhibition of GABA^RVM^ neurons.

**Graphical Abstracts:**

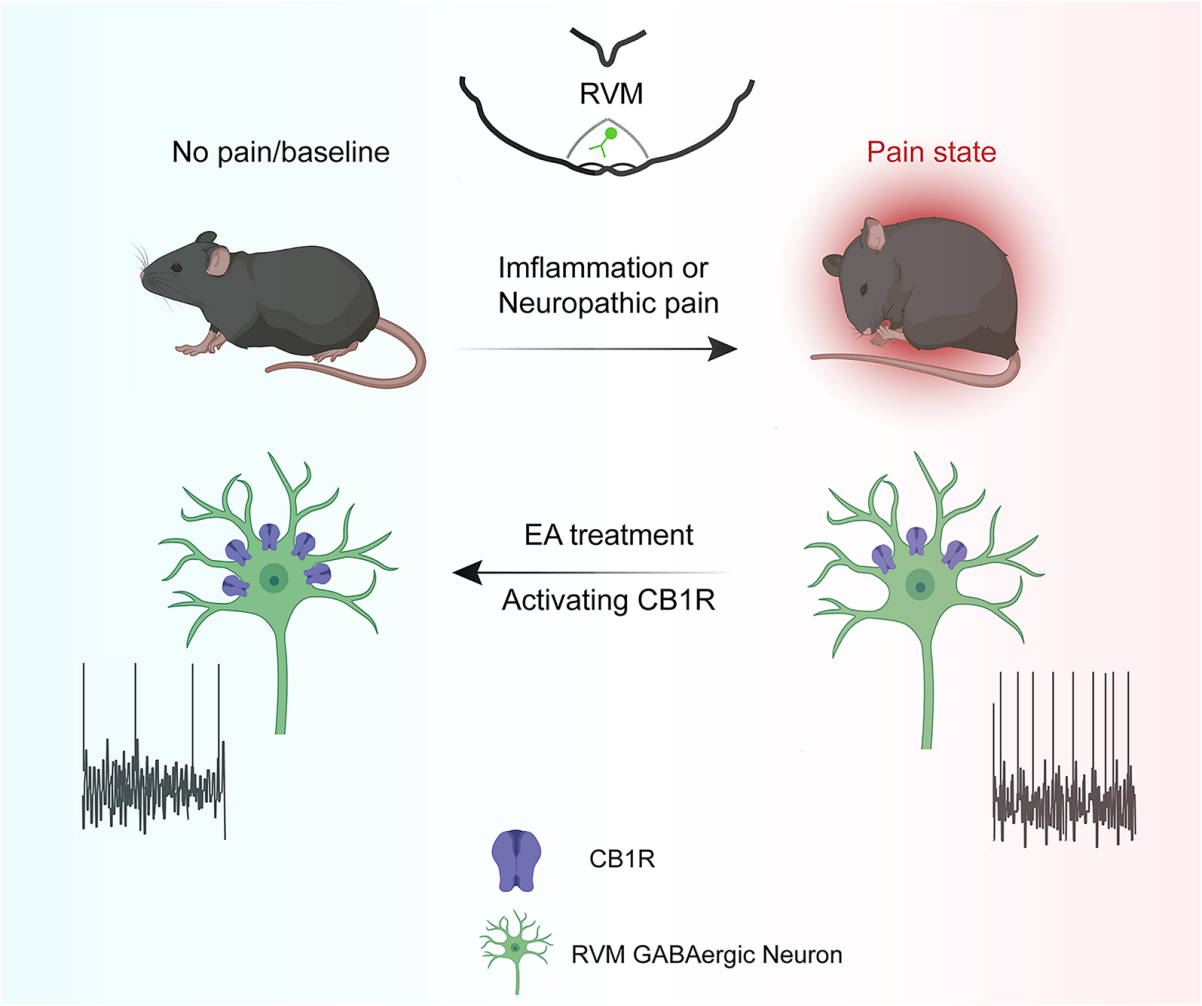

**Supplementary Information:**

The online version contains supplementary material available at 10.1186/s13020-025-01083-4.

## Introduction

Pain is a subjective feeling and response of the body to noxious stimuli [1]. At the initial stage of pain, it usually manifests as acute pain, which is a protective mechanism of the body, reminding people to avoid further harm and prompting the body to repair itself [2]. If acute pain is not treated promptly and effectively, or noxious stimuli persist, the pain may develop into chronic pain [3]. Chronic pain lasts for a long time, and can be caused by many reasons, such as nerve injury and inflammation [4]. In the state of chronic pain, a series of plastic changes will occur in the nervous system, which will affect the quality of life of patients such as mood, sleep, diet and social interaction, further aggravate the psychological burden, and make the pain problem more complicated and difficult to treat [5]. Chronic pain has a significant personal and economic impact, affecting over 30% of the world's population [6–8]. Current medications have limited analgesic effects and often produce various adverse effects [9, 10]. Consequently, identifying safer and more effective pain treatments is urgently needed. Acupuncture, a traditional therapy with a long history of use in China, has been endorsed by the World Health Organization for treating 77 different diseases [11]. Among the various applications of acupuncture, pain management stands out as a major therapeutic benefit [12]. Electroacupuncture (EA), a modified form of acupuncture introduced in the mid-twentieth century, combines traditional acupuncture techniques with a mild electric current to effectively reduce both acute and chronic pain [13, 14]. EA is commonly used for pain control due to its perceived safety, efficacy, and minimal side effects [15]. The exact mechanisms underlying the analgesic effect of EA remain incompletely understood.

The rostral ventromedial medulla (RVM) is a region located in the brainstem, which includes the nucleus raphe magnus (NRM) and the reticular nucleus of the giant cell (NRGc), plays a crucial role in the endogenous pain modulation system [16]. It is involved in both the facilitation and inhibition of pain as part of the descending pain modulation system [17]. [18] The RVM contains various types of neurons, including serotonergic (5-HT), GABAergic, and glutamatergic neurons, which contribute to the complex regulation of pain signals [19]. RVM neurons are also classified into on-cells, off-cells, or neutral cells based on their responses to painful stimuli [20]. These neurons, via myelinated fibers, play a crucial role in pain regulation by projecting to the spinal cord [21].

It's reported that GABA neurons account for a large proportion in RVM brain region, 27.27% of RVM On cells, 47.37% of RVM Off cells, and 42.6% of RVM Neutral cells were GABAergic [22]. GABAergic neurons play an important inhibitory role in the central nervous system, especially in pain regulation [23]. They inhibit the activity of other neurons by releasing γ-aminobutyric acid (GABA), thus maintaining the balance of neural network [24]. Studies have shown that the hyperactivity of GABAergic neurons in the RVM (GABA^RVM^ neurons) may lead to increased pain sensitivity, which is closely related to the activity of off-cells [25]. Off-cells are mainly responsible for inhibiting the transmission of pain signals, and activation of GABA^RVM^ neurons can effectively reduce the excitability of off-cells through their inhibition effects, thus participating in the facilitation of descending pain [26].

The cannabinoid CB1 receptor (CB1R), known for its role in regulating motor function, pain, and psychoactive actions, is expressed in various brain regions [27]. CB1R is known to be expressed in both presynaptic and postsynaptic synapses [28]. However, it mainly found at presynaptic terminals and is predominantly expressed in GABAergic neurons, with lower levels in glutamatergic neurons [29]. Activating CB1Rs in the RVM can directly reduce neurotransmitter release from GABA neuronal terminals [30, 31]. Additionally, EA can activate CB1Rs, leading to alleviation of painful conditions [29, 32–34]. The role of CB1 receptors on GABA^RVM^ neurons in mediating the analgesic effects of EA remains largely uncertain.

This study utilized inflammatory pain models induced by complete Freund's adjuvant (CFA) and neuropathic pain models from chronic constrictive injury (CCI) to evaluate the involvement of CB1Rs and GABA^RVM^ neurons in the analgesic effects of EA. Our findings highlight the crucial involvement of CB1Rs and GABAergic neurons in the RVM in mediating the analgesic effects of EA.

## Materials and methods

### Animals

The Institutional Animal Care and Use Committee (IACUC) at Huazhong University of Science and Technology approved all experimental protocols. Male mice, aged between 8 and 10 weeks and weighing 20 to 30 g, were employed in this research. The study employed Vgat-ires-cre mice (STOCK Slc32a1tm2(cre)Lowl/J) (JAX016962), CB1B flox/flox mice (Shanghai Model Organisms Center, Inc.), and wild-type C57BL/6 J mice (Beijing Vital River Laboratory Animal Technology Co., Ltd.). Animals were allocated to various experimental groups at random. The subjects were housed in cages under a 12-h light/dark cycle with unrestricted access to food and water. The mice were kept in a controlled environment with temperature ranged from 22 °C to 25 °C and humidity maintained at 50% ± 10%. The mice were kept in cages located on the same rack.

### Pain models

To induce prolonged inflammatory pain, 25 μL of CFA was administered into the left hind paws of mice under brief isoflurane anesthesia [35]. Control mice received the same volume of saline administered in the same manner.

The CCI-induced neuropathic pain model was implemented according to the established protocol [36]. The mice were anesthetized with 2–3% isoflurane, and the left sciatic nerve was exposed at the mid-thigh. At the branching point, the nerve trunk was lightly tied three times using 4–0 gut sutures. Sham mice experienced the same procedures but without constricting the nerve.

### EA treatment

EA on the ST36 acupoint can alleviate inflammatory pain through diverse mechanisms, while the parameters of EA stimulation are not uniform [37]. A recent study found that weak EA stimulation at the ST36 point with 0.5 mA/10 Hz effectively activated the vagal-adrenal axis, leading to the suppression of systemic inflammatory responses [38]. To enhances the understanding of optimal acupoint selection and stimulation intensity for EA analgesic therapy, so we choose EA stimulation at the ST36 point with 0.5 mA/10 Hz for the test. Using homemade clothing (20 cm × 15 cm), the mice were carefully restrained, allowing their legs to protrude through the openings. Two 0.25 mm × 13 mm acupuncture needles (Beijing Zhongyan Taihe Medical Instruments Co., Ltd., China) were inserted 5 mm into the region and connected to an 8-channel stimulus generator (STG4008, Reutlingen, Germany). EA treatment was applied for ipsilateral ST36 (ST36 was situated near the knee joint under the lateral, fibula 3.5 mm below the capitulum) at 0.5 mA intensity and 10 Hz frequency for 30 min daily [38]. During the procedure, the animals remained awake, immobile, and showed no obvious signs of distress. The control group received only restraint manipulations.

### Behavior tests

Behavioral assessments, including the measurement of nociceptive thresholds, open field test, conditioned place preference test, and conditioned place aversion test, were carried out as specified in the Supplementary Materials.

### Western blot analysis

CB1R protein levels were assessed using Western blotting. Protein lysates were obtained from RVM tissues. For detailed methods, refer to the Supplementary materials.

### Viral microinjection, in vivo calcium imaging via fiber photometry, and electrophysiological recordings in vivo

Comprehensive methodologies for viral microinjection, optical fiber implantation, fiber photometry for in vivo calcium imaging, and in vivo electrophysiological recordings was carried out according to the previous research [39, 40]. Details are provided in the Supplementary materials.

### RNAscope in situ hybridization

The overlap of GABA and c-Fos in the RVM was assessed using the RNAscope multiplex fluorescent reagent kit v2 and specifically designed probes (ACD Bio Inc.). Mice were anesthetized with isoflurane and perfused transcardially with 25 ml PBS and 25 ml 4% paraformaldehyde. Following perfusion, the brain was isolated and post-fixed overnight at 4 °C with the same fixative. Tissues were washed multiple times in PBS, and then cryopreserved with a sucrose gradient. Tissues were embedded in OCT medium (Tissue-Tek), cryosectioned into 14 μm sections, and mounted on charged slides. We used probes directed against mouse *fos* (catalog 316921) and mouse *Slc32a1* (a marker of GABAergic neurons; catalog 319191). In situ hybridization was performed using the RNAscope system (Advanced Cell Diagnostics) following the manufacturer’s instructions with the Multiplex Fluorescent Kit v.2 protocol.

### Image acquisition and quantification

Samples were imaged on an Olympus VS-200 laser scanning microscope. Samples for immunohistochemical staining were imaged at 10 × magnification. Neurons and Fos-expressing neurons identified through in situ hybridization were manually counted, with percentages determined for each animal and averaged across the group. We assessed relative marker expression by aggregating cell counts from brain sections of 3 to 4 mice.

### Statistical analysis

The investigator performing data analysis was blinded to the group allocation. GraphPad Prism 9.0 was used for the statistical analysis. Group differences were evaluated using the Kruskal–Wallis test, with Dunn's post hoc test applied for data not following a normal distribution. For normally distributed data, analyses included two-tailed unpaired t-tests and one-way or two-way ANOVA, followed by Tukey or Bonferroni post hoc tests. Graphical data are presented as means with standard error of the mean (SEM). A difference is considered statistically significant if the p-value is less than 0.05.

## Results

### EA produces analgesic effects and relieves the negative emotion caused by pain in CFA- and CCI-treated mice

**Fig. 1:**
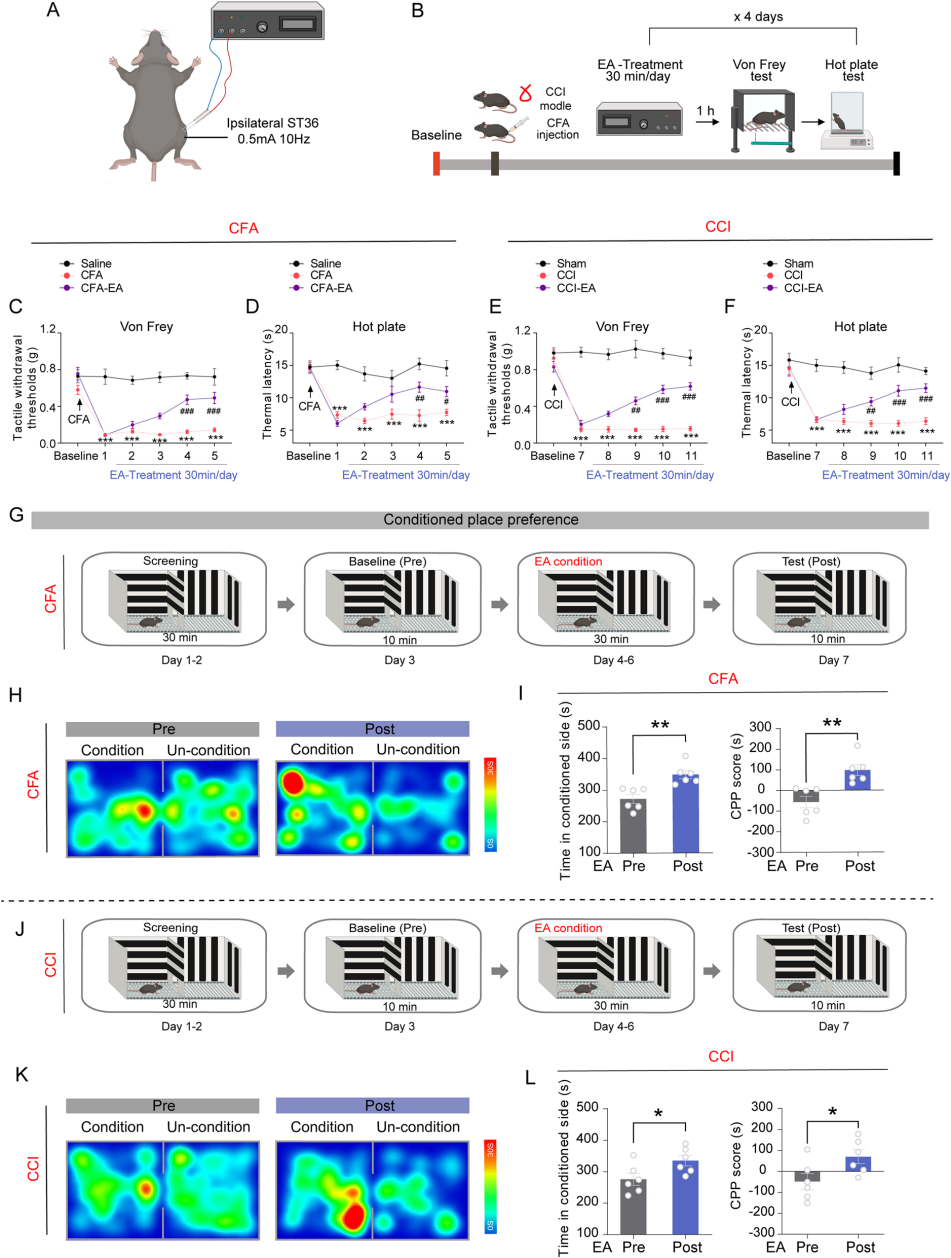
EA produces analgesia and negative emotion-relieving effects in mice with inflammatory and neuropathic pain. **A** The schematic diagram of EA treatment of ipsilateral ST36 acupoints of the affected side in mice at an intensity of 0.5 mA/10 Hz; **B** Experimental design and timeline of the behavioral experiment. The panels A and B were generated with BioRender (https://biorender.com/). **C** Time course of changes in the tactile withdrawal thresholds based on von Frey tests in the CFA model. **D** Time course of changes in the thermal withdrawal latency based on hot plate test in CFA model. **E** Time course of changes in the tactile withdrawal thresholds based on von Frey tests in the CCI model. **F** Time course of changes in the thermal withdrawal latency based on hot plate test in CCI model. For C-F, *** *p* < 0.001 vs. Saline or Sham control group, ^#^ *p* < 0.05, ^##^ *p* < 0.01 and ^###^ *p* < 0.001 vs. CFA or CCI group, revealed by two-way ANOVA with Bonferroni’s post hoc test, n = 6 mice per group. **G** The schematic diagram of the experiment design for the conditioned place preference (CPP) test. **H** The representative tracking maps of CFA mice in the CPP before and after the EA condition. **I** The time spent in the EA condition chamber and CPP score of CFA-treated mice.** J** The schematic diagram of the experiment design for the conditioned place preference (CPP) test. **K** The representative tracking maps of CCI mice in the CPP before and after the EA condition. **L** The time spent in the EA condition chamber and CPP score of CCI-treated mice. For I and L, *p < 0.05, **p < 0.01 vs. pre-EA condition, revealed by two-tailed paired t-test, n = 6 mice per group. All data are shown as mean ± SEM

Tactile allodynia was present in all mice receiving CFA or CCI (Fig. 1). EA was administered at the Zusanli acupoints (ST36, 0.5 mA/10 Hz) for 30 min daily 2 to 5 days after CFA, or 8 to 11 days after CCI (Fig. 1A, B). Behavioral assessment showed that CFA and CCI significantly lowered paw withdrawal thresholds in mice when exposed to von Frey and heat stimuli (Fig. 1C–F, p < 0.001). In addition, the ipsilateral EA has a better analgesic effect than opposite EA, so we used ipsilateral EA treatment in the following study (supplementary Fig. 1). EA elevated tactile withdrawal thresholds and thermal withdrawal latency on days 4–5 in CFA-injected mice and days 9–11 in CCI-exposed mice (Fig. 1C–F, p < 0.05). We performed CPP experiments to assess the impact of EA on negative emotions in mice. The findings indicated that the time spent by CFA- or CCI-treated mice in the chamber matched EA treatment and the CPP score exhibited a significant increase compared to the baseline measurements (Fig. 1G–L, p < 0.05).

### EA suppresses GABARVM neuronal activity in CFA- and CCI-treated mice

**Fig. 2:**
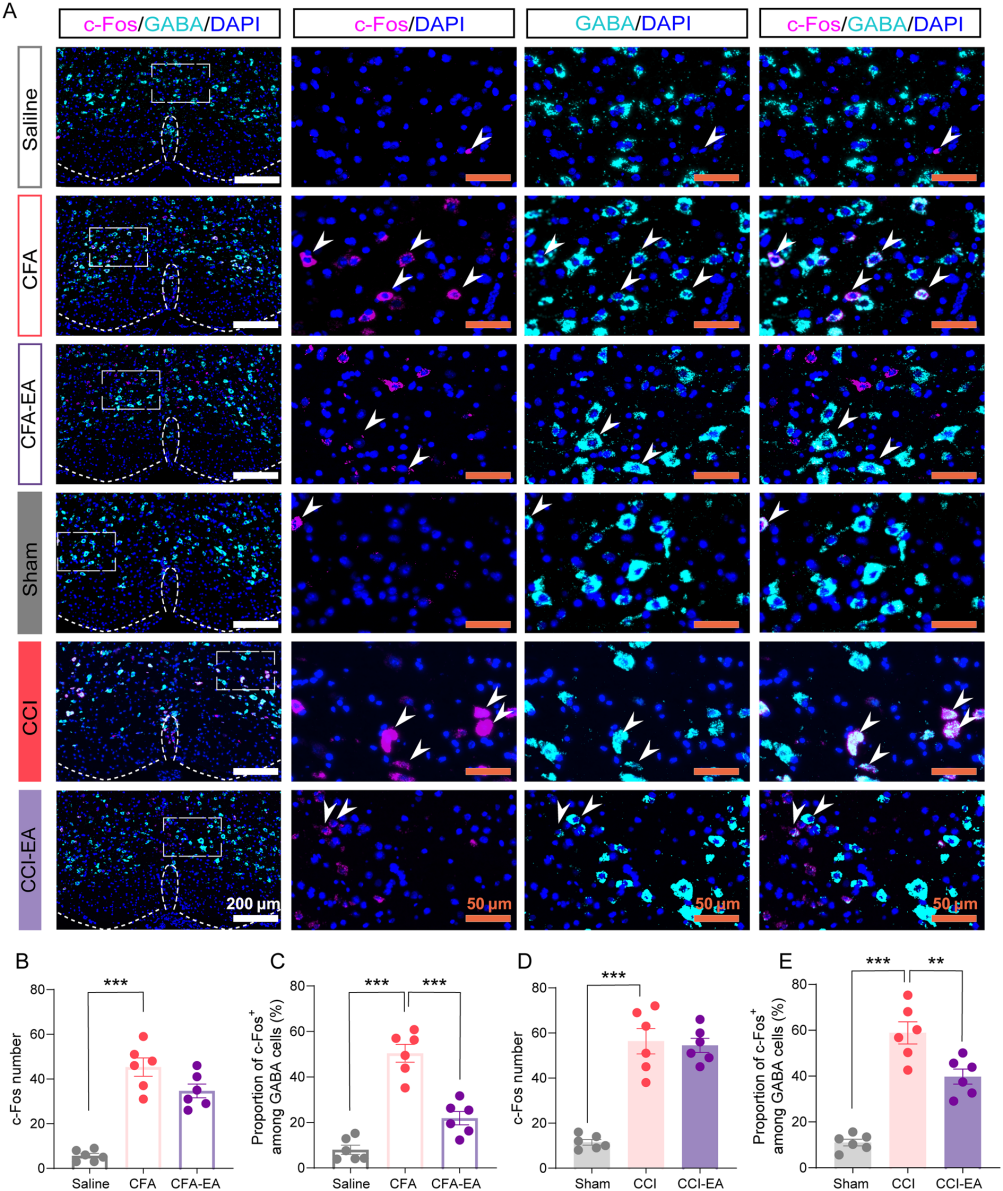
EA reduces c-Fos-positive GABA^RVM^ neurons in CFA- and CCI-treated mice. **A** Representative in situ hybridization images for the GABA (green, Slc32a1)- and c-Fos (red)-labeled cells in the RVM. Scale bars = 200 μm. Right panels, 4 × magnification of the boxed area in the left panel, Scale bars = 50 μm. **B** The number of c-Fos-positive cells in the RVM of Saline, CFA, and CFA-EA groups. **C** The proportions of GABA-positive cells co-expressing c-Fos in the RVM of Saline, CFA, and CFA-EA groups. **D** The number of c-Fos-positive cells in the RVM of Sham, CCI, and CCI-EA groups. **E** The proportions of GABA-positive cells co-expressing c-Fos in the RVM o of Sham, CCI, and CCI-EA groups. **p < 0.01 and ***p < 0.001 vs. CFA or CCI group, one-way ANOVA followed by Dunnett’s post hoc tests, n = 6 sections from 3 mice per group. All data are shown as mean ± SEM

To determine GABA^RVM^ neuronal activity associated with pain hypersensitivity and the EA treatment effect, we conducted in situ hybridization, calcium imaging in living mice, and in vivo electrophysiological techniques. RNAscope in situ hybridization revealed a significant increase in c-Fos positive cells in the RVM of CFA and CCI mice compared to control mice (Fig. 2A–E, p < 0.001). EA treatment showed no impact on the overall count of c-Fos positive cells in both CFA and CCI mice (Fig. 2A–B, D, p > 0.05). Co-labeling of c-Fos and Slc32a1, indicative of GABAergic neurons, showed a significant increase in c-Fos positive GABAergic cells in CFA and CCI mice, which was mitigated by EA treatment (Fig. 2A, C, E, p < 0.01).

**Fig. 3:**
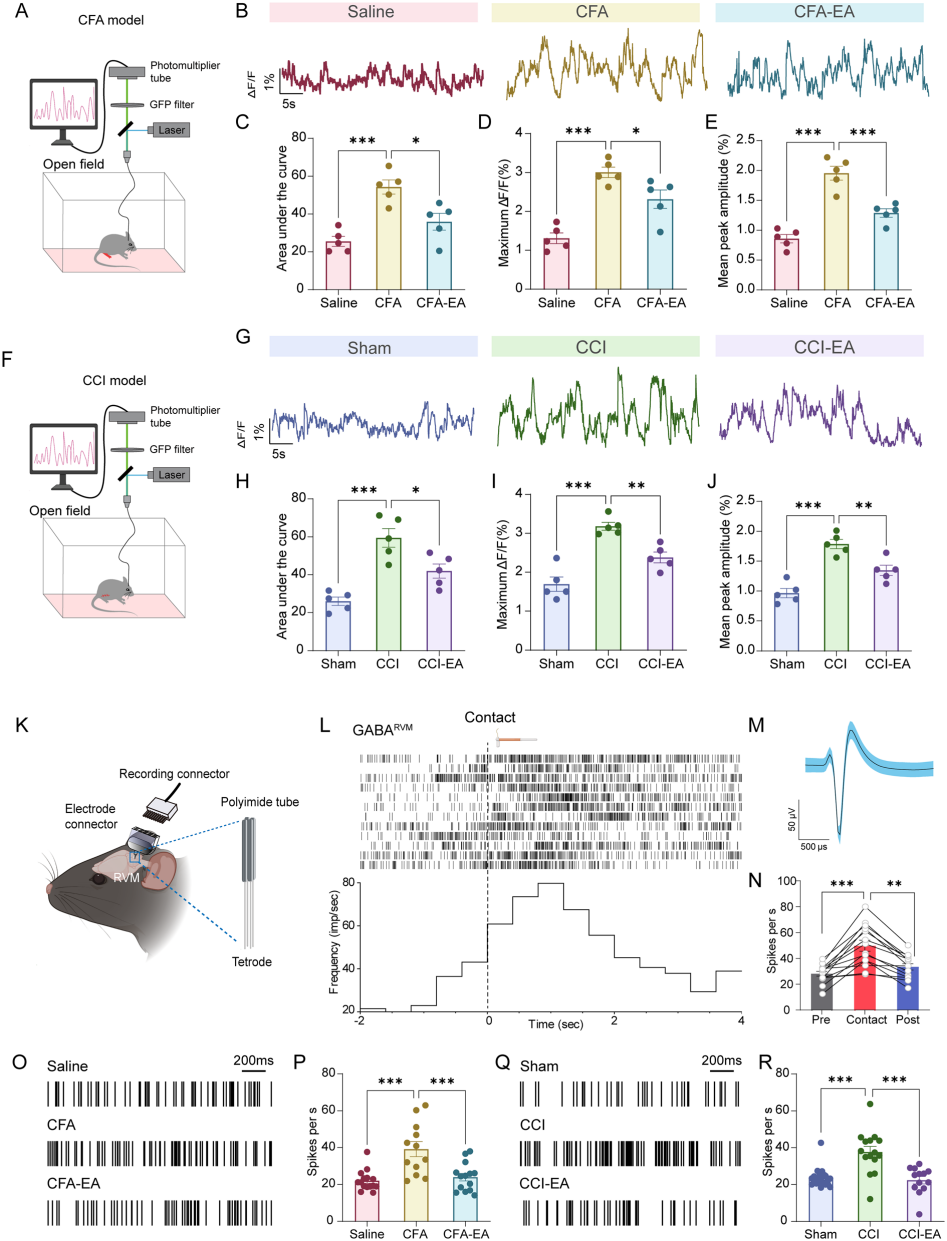
EA suppresses GABA^RVM^ neuronal activity in CFA- and CCI-treated mice. **A** Experimental design for recording GCaMP6s fluorescence signals from GABA^RVM^ neurons of CFA-treated mice. **B** Representative GCaMP6s fluorescence signals recorded from the Saline (left), CFA (middle), and CFA-EA (right) groups. **C**-**E** The area under the curve (AUC) (**C**), maximum ΔF/F (**D**), and mean peak amplitude (**E**) of GCaMP6s fluorescence signals were recorded from the Saline, CFA, and CFA-EA groups. **F** Experimental design for recording GCaMP6s fluorescence signals from GABA^RVM^ neurons of CCI-treated mice. **G** Representative GCaMP6s fluorescence signals were recorded from the Sham (left), CCI (middle), and CCI-EA (right) groups. **H-J** The area under the curve (AUC) (H), maximum ΔF/F (**I**), and mean peak amplitude (**J**) of GCaMP6s fluorescence signals recorded from the Sham, CCI, and CCI-EA groups. For **C**-**E** and **H**-**J**, significance levels are indicated as * *p* < 0.05, ** *p* < 0.01 and *** *p* < 0.001 compared to the CFA/CCI group, using one-way ANOVA with Dunnett’s post hoc test, n = 5 mice per group. **K** A schematic representation of electrophysiological recordings within the RVM in freely moving mice. The enlarged section highlights the multichannel tetrode. **L** The displayed are the raster plot (upper part) and the peri-stimulus time histogram (lower part) illustrating the multi-channel recordings of GABA^RVM^ neuronal firing rates in control mice before, during, and following von Frey (1.0 g) stimuli (n = 12 cells from 2 mice per group). **M** Overlay of contact-evoked (blue) and averaged spontaneous (navy Blue) spike waveforms from the example unit. **N** The firing rates of GABA^RVM^ neurons in control mice before, during, and after von Frey (1.0 g) stimuli, ** *p* < 0.01 and *** *p* < 0.001 vs. Contact group, repeated measures one-way ANOVA followed by Dunnett’s post hoc test, n = 12 cells from 3 mice per group. **O** Representative recording of spontaneous spikes and data **P** showing GABA^RVM^ neuronal firing rates in mice treated with saline, CFA, and CFA-EA (n = 15 cells from 3 mice for RVM::Saline; n = 12 cells from 3 mice for RVM::CFA; n = 14 cells from 3 mice for RVM::CFA-EA). **Q** Representative recording of spontaneous spikes and data **R** showing GABA^RVM^ neuronal firing rates in mice treated with Sham, CCI, and CCI-EA (n = 17 cells from 3 mice for RVM:: Sham; n = 15 cells from 3 mice for RVM:: CCI; n = 13 cells from 3 mice for RVM:: CCI-EA). Scale bars = 200 ms, ***p < 0.001 vs. CFA or CCI group, one-way ANOVA followed by Dunnett’s post hoc tests. All data are shown as mean ± SEM

Calcium imaging in live mice revealed a significant increase in the GCaMP signal from GABA^RVM^ neurons in CFA- and CCI-treated mice compared to the control group, as indicated by a higher area under the curve (AUC), greater maximum change in fluorescence (dF/F), and elevated mean peak amplitude (Fig. 3A–J, p < 0.001). Furthermore, applying a noxious stimulus (pinch) and a mechanical stimulus (1.0 g von Frey) to the hindpaw elicited measurable Ca^2+^ responses in control mice (Saline and Sham groups). These reactions were notably higher in mice that underwent CFA and CCI (supplementary Fig. 2).

EA treatment significantly decreased the GCaMP signal in GABA^RVM^ neurons of mice treated with CFA and CCI (Fig. 3A–J, p < 0.05). These results were confirmed by in vivo electrophysiological recordings. Nociceptive stimuli increased the firing rate of GABA^RVM^ neurons of control mice (Fig. 3K–N, p < 0.001). Also, the firing rate of GABA^RVM^ neurons was elevated in mice subjected to CFA and CCI, and EA treatment reduced the neuronal firing activity of GABA^RVM^ neurons in CFA- or CCI-treated mice (Fig. 3O–R, p < 0.001). These data suggest that EA at ipsilateral ST36 suppresses GABA^RVM^ neuronal activity augmented by tissue inflammation and nerve injury.

### Inhibition of GABARVM neurons alleviates pain-like hypersensitivity induced by CFA and CCI

**Fig. 4:**
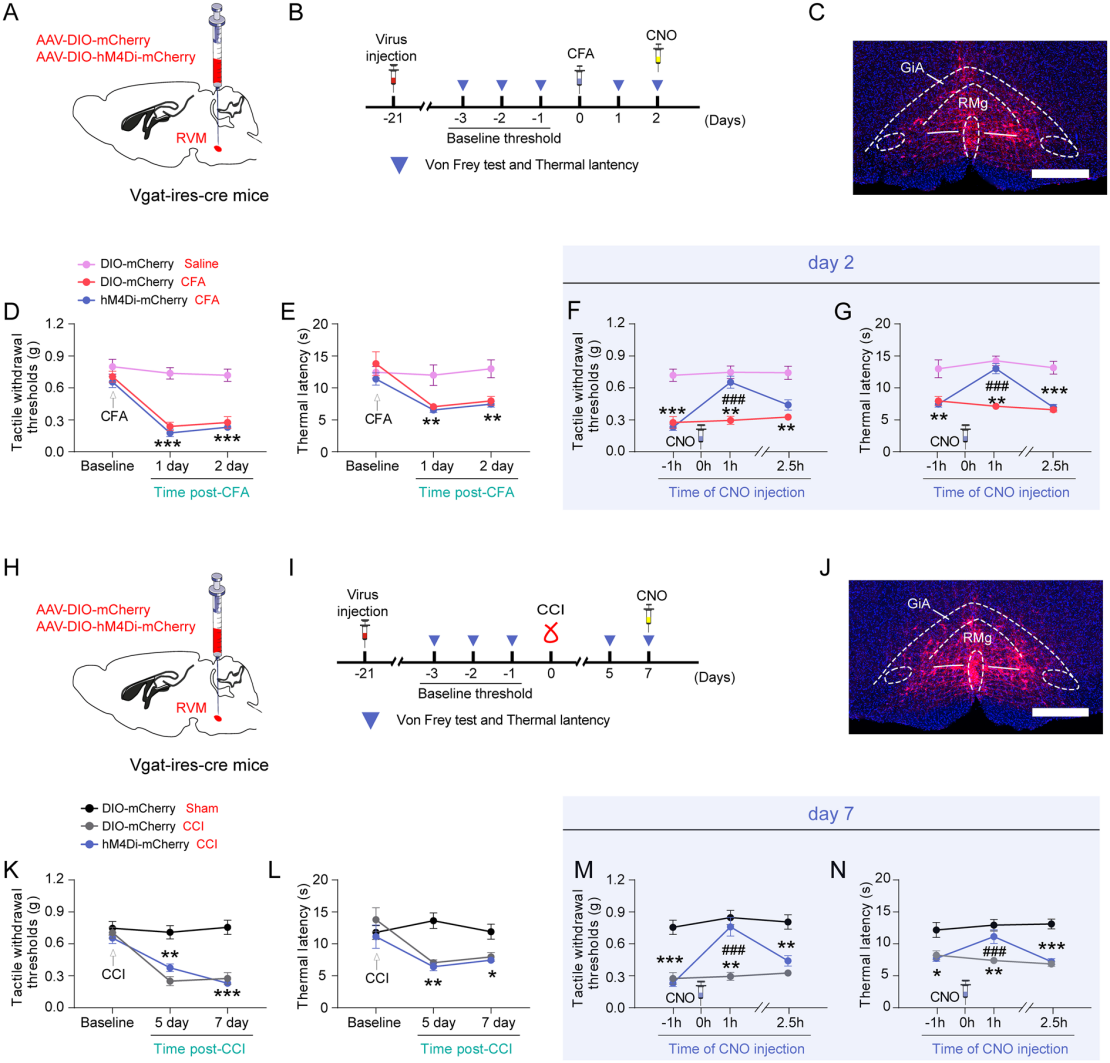
Inhibition of GABA^RVM^ neuron alleviates pain hypersensitivity induced by CFA and CCI. **A** The diagram illustrating the stereotaxic injection of AAV-DIO-hM4Di-mCherry or AAV-DIO-mCherry viruses into the RVM region of vgat-ires-cre mice. **B** The experimental design and timeline of the behavioral tests for inhibiting GABA^RVM^ neurons in the CFA model. **C** The representative fluorescence image shows the injection site of the virus. Scale bars = 500 μm. **D-E** The time course of changes in the tactile withdrawal thresholds (**D**) and the thermal withdrawal latency (**E**) in the vgat-cre::DIO-mCherry and vgat-cre::DIO-hM4Di mice after CFA treatment. **F-G** The time course of changes in the tactile withdrawal thresholds (**F**) and the thermal withdrawal latency (**G**) in CFA-treated vgat-cre::DIO-mCherry and vgat-cre::DIO-hM4Di mice after CNO injection. **H** The diagram illustrating the stereotaxic injection of AAV-DIO-hM4Di-mCherry or AAV-DIO-mCherry viruses into the RVM region of vgat-ires-cre mice. **I** The experimental design and timeline of the behavioral tests for inhibiting GABA^RVM^ neurons in the CCI mice. **J** The representative fluorescence image shows the injection site of the virus. Scale bars = 500 μm. **K-L** The time course of changes in the tactile withdrawal thresholds (**K**) and the thermal withdrawal latency (**L**) in the vgat-cre::DIO-mCherry and vgat-cre::DIO-hM4Di mice after CCI treatment. **M–N** The time course of changes in the tactile withdrawal thresholds (**M**) and the thermal withdrawal latency (**N**) in the CCI-treated vgat-cre::DIO-mCherry and vgat-cre::DIO-hM4Di mice after CNO injection. **p < 0.01 and ***p < 0.001 vs. Saline or Sham control group, ^###^*p* < 0.001 vs. CFA- or CCI-DIO-mCherry group, two-way ANOVA followed by Bonferroni post hoc tests, n = 6 mice per group. All data are shown as mean ± SEM

We injected the AAV-DIO-mCherry-hM4Di virus into the RVM of Vgat-ires-cre mice to assess the role of GABA^RVM^ neuronal activity in pain hypersensitivity caused by CFA and CCI (Fig. 4A–N). Behavioral assessment showed that on days 1–2 after CFA injection or days 5–7 after CCI induction, the mice exhibited a notable decrease in their tactile withdrawal thresholds and thermal paw withdrawal latency (Fig. 4D–E, K–L, p < 0.05). Chemogenetic inhibition of GABA^RVM^ neurons elevated tactile withdrawal thresholds to von Frey stimulation and increased paw withdrawal latency to thermal stimulation in mice treated with CCI and CFA (Fig. 4F–G, M–N, p < 0.001). After a 2.5-h washout period of CNO, the tactile withdrawal thresholds and thermal paw withdrawal latency returned to baseline levels (Fig. 4F–G, M–N). These findings suggest that increased GABA^RVM^ neuronal activity mediates both inflammation- and nerve injury-induced pain hypersensitivity. Moreover, GABA^RVM^ neuronal activity plays a role in basal mechanical nociception but not thermal nociception, locomotor and anxiety levels in control mice (supplementary Fig. 3).

### GABARVM neuron activation counteracts the analgesic effect of EA

**Fig. 5:**
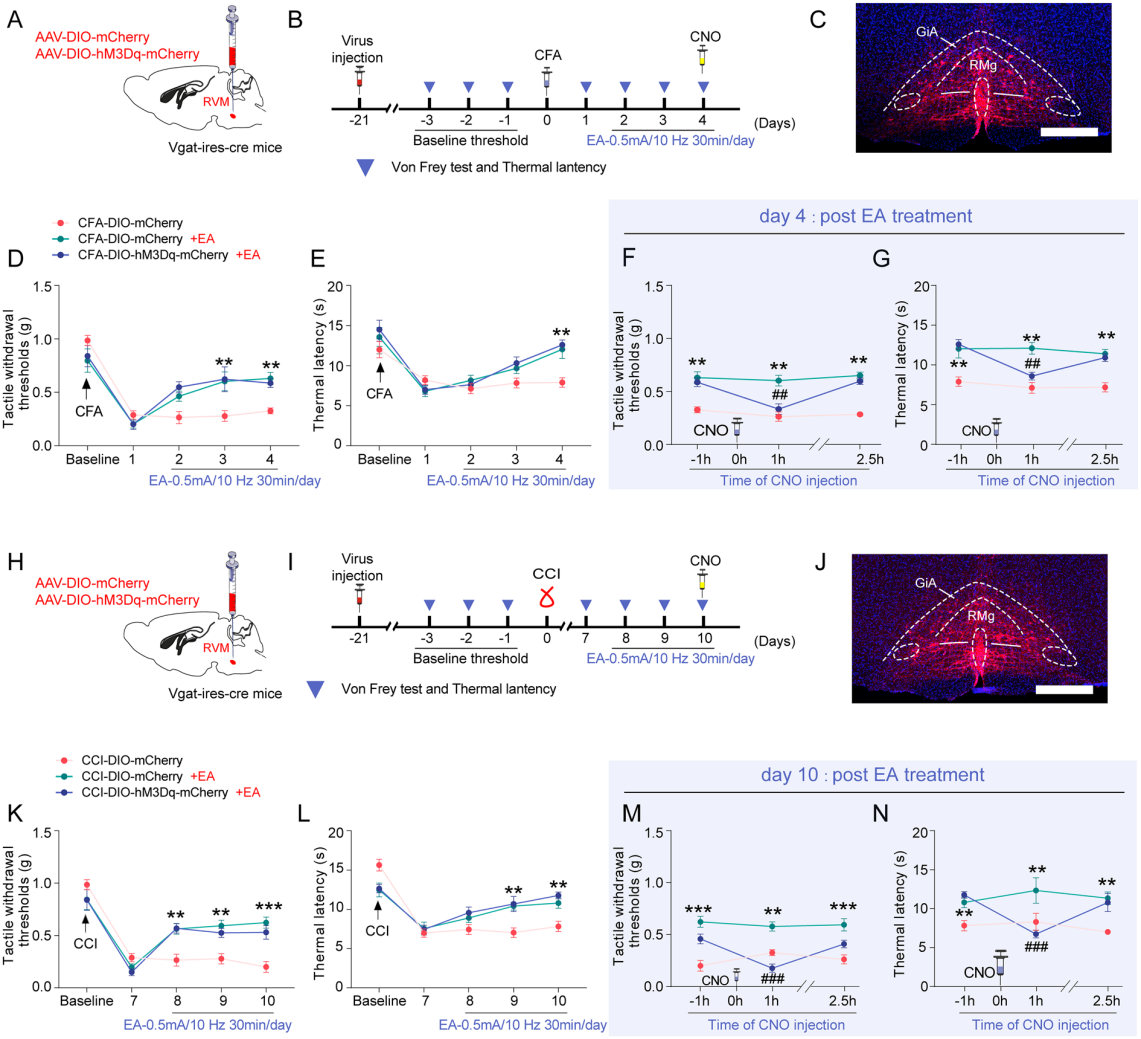
GABA^RVM^ neuron activation diminishes EA's analgesic efficacy. **A** The diagram illustrating the stereotaxic injection of AAV-DIO-hM3Dq-mCherry or AAV-DIO-mCherry viruses into the RVM region of vgat-ires-cre mice. **B** The experimental design and timeline of the behavioral experiment for activating GABA^RVM^ neurons in the CFA model. **C** The representative fluorescence image shows the injection site of the virus. Scale bars = 500 μm. **D-E** The time course of EA treatment effect on the tactile withdrawal thresholds (**D**) and the thermal withdrawal latency (**E**) in vgat-cre::DIO-mCherry and vgat-cre::DIO-hM3Dq mice treated with CFA. **F-G** The time course of the effects of EA and CNO treatment on the tactile withdrawal thresholds (**F**) and thermal withdrawal latency (**G**) in vgat-cre::DIO-mCherry and vgat-cre::DIO-hM3Dq mice treated with CFA. **H** The diagram illustrating the stereotaxic injection of AAV-DIO-hM3Dq-mCherry or AAV-DIO-mCherry viruses into the RVM region of vgat-ires-cre mice. **I** The experimental design and timeline of the behavioral experiment for activating GABA^RVM^ neurons in CCI mice. **J** The representative fluorescence image shows the injection site of the virus. Scale bars = 500 μm. **K-L** The time course of EA treatment effect on CCI-induced tactile withdrawal thresholds (**K**) and the thermal withdrawal latency (**L**) in vgat-cre::DIO-mCherry and vgat-cre::DIO-hM3Dq mice. **M–N** The time course of effects of EA and CNO treatment changes on CCI-induced tactile withdrawal thresholds **M** and the thermal withdrawal latency **N** in vgat-cre::DIO-mCherry and vgat-cre::DIO-hM3Dq mice. *p < 0.05, **p < 0.01 and ***p < 0.001 vs. CFA or CCI-DIO-mCherry group, ^#^*p* < 0.01 and ^###^*p* < 0.001 vs. CFA- or CCI-DIO-mCherry + EA group, two-way ANOVA followed by Bonferroni’s post hoc tests, n = 6 mice per group. All data are shown as mean ± SEM

To assess the impact of GABA^RVM^ neuron activation on EA analgesia, we administered the AAV-DIO-hM3Dq-mCherry virus into the RVM of Vgat-ires-Cre mice subjected to CCI and CFA induction (Fig. 5A–N). Behavioral tests showed that on 2–3 days after EA-treatment, both CFA- and CCI-treated mice exhibited a notable increase in their tactile withdrawal thresholds and thermal withdrawal latency (Fig. 5D–E, K–L, p < 0.05). Chemogenetic activation of GABA^RVM^ neurons inhibited the impact of EA on tactile withdrawal thresholds and thermal withdrawal latency in mice treated with CCI and CFA (Fig. 5F–G, M–N, p < 0.01). After a 2.5-h washout period of CNO, the tactile withdrawal thresholds and thermal withdrawal latency reverted to the baseline levels (Fig. 5F–G, M–N). The results indicate that activating GABA^RVM^ neurons counteracts the analgesic effect of EA. Activation of GABA^RVM^ neurons induces mechanical and thermal pain hypersensitivity in control mice without altering locomotor or anxiety levels (supplementary Fig. 4).

### Knockdown of CB1Rs on GABARVM neurons antagonizes the analgesic and negative mood-relieving effects of EA

**Fig. 6:**
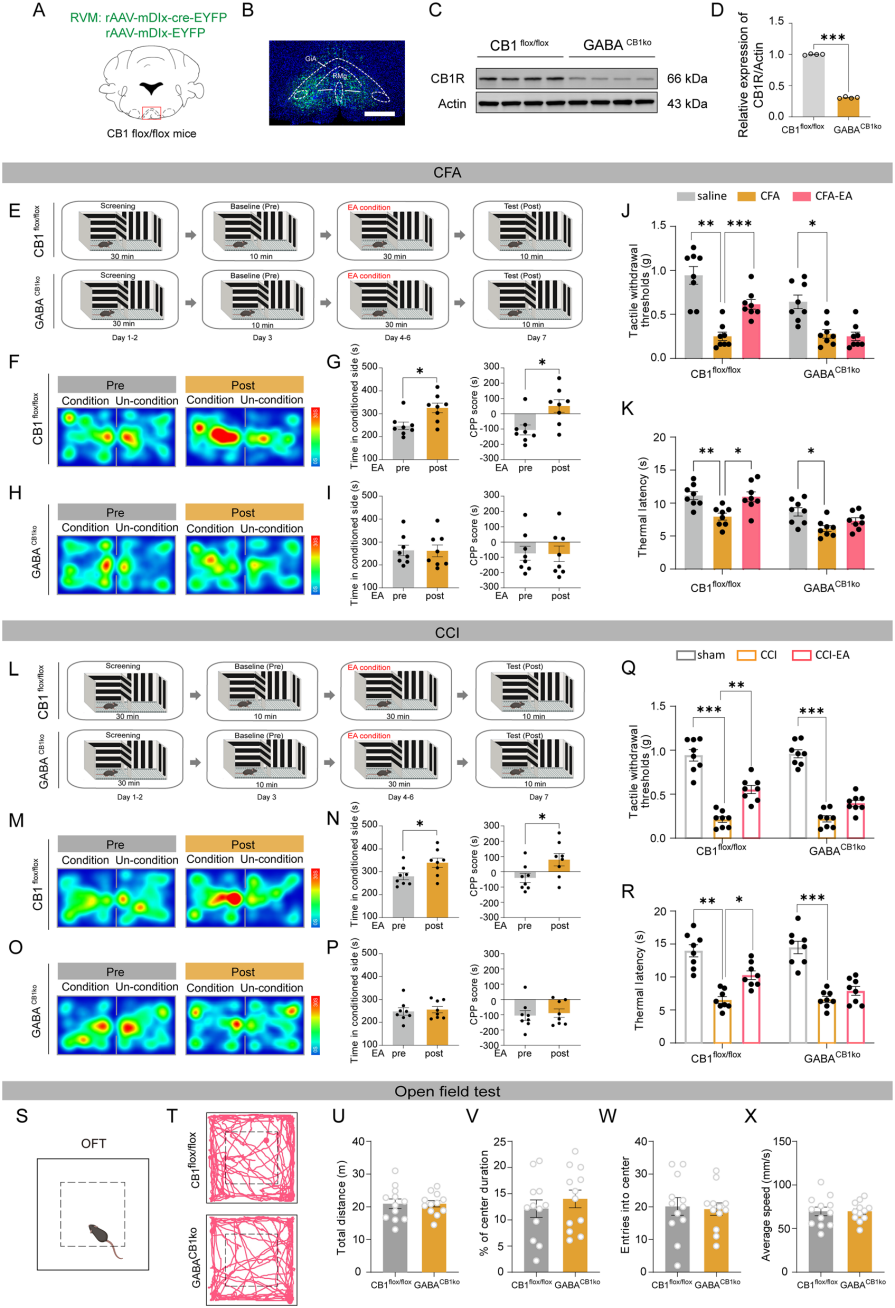
Knockdown of CB1Rd on GABA^RVM^ neurons antagonizes the analgesic and negative mood-relieving effects of EA. **A** The diagram illustrating the stereotaxic injection of AAV-mDlx-cre-EYFP virus or AAV-mDlx-EYFP viruses into the RVM region of CB1^flox/flox^ mice. **B** The representative fluorescence image shows the injection site of the virus. Scale bars = 500 μm. **C** Representative immunoblots of CB1R protein levels in the RVM. **D** The CB1R expression levels were normalized to the expression level of β-actin, n = 4 mice per groups, *** *p* < 0.001 vs. CB1^flox/flox^ control group, two-tailed unpaired t-test. **E** The schematic diagram of the experiment design for the conditioned place preference (CPP) test. **F** The representative tracking maps of CFA-induced CB1^flox/flox^ mice in the CPP before and after the EA conditioning. **G** The time spent in the EA-conditioned chamber and CPP score of CFA-treated CB1^flox/flox^ mice. **H** The representative tracking maps of CFA-treated GABA^CB1KO^ mice in the CPP before and after the EA conditioning. **G** The time spent in the EA-conditioned chamber and CPP score of CFA-treated GABA^CB1KO^ mice. For G and I, *p < 0.05 vs. pre-EA condition, two-tailed paired t-test, n = 8. **(J-K)** The effect of CFA and EA on the tactile withdrawal thresholds (J) and the thermal withdrawal latency (K) of CB1^flox/flox^ and GABA^CB1KO^ mice. **L** The schematic diagram of the experiment design for the conditioned place preference (CPP) test. **M** The representative tracking maps of CCI-treated CB1^flox/flox^ mice in the CPP before and after the EA conditioning. **N** The time spent in the EA-conditioned chamber and CPP score of CCI-treated CB1^flox/flox^ mice. **O** The representative tracking maps of CCI-treated GABA^CB1KO^ mice in the CPP before and after the EA conditioning. **P** The time spent in the EA conditioned chamber and CPP score of CCI-treated GABA^CB1KO^ mice. For **G** and **I**, *p < 0.05 vs. pre-EA condition, two-tailed paired t-test, n = 8 mice per group. **Q-R** The effect of CCI and EA on the tactile withdrawal thresholds (**Q**) and the thermal withdrawal latency (R) of CB1^flox/flox^ and GABA^CB1KO^ mice. For J-K and Q-R, *p < 0.05, **p < 0.01 and ***p < 0.001 vs. CFA- or CCI-treatment within CB1^flox/flox^ and GABA^CB1KO^ group, two-way ANOVA followed by Bonferroni’s post hoc tests, n = 8 mice per group. **S-T** The representative tracking maps of CB1^flox/flox^ and GABA^CB1KO^ mice in the open field test. **U-X** The total distance moved (**U**), the ratio of time spent in the center (**V**), the entries into the center (**W**), and the average speed (**X**) of CB1^flox/flox^ and GABA^CB1KO^ mice in the open field. n = 12 mice per group. All data are shown as mean ± SEM

CB1 receptor activation in the RVM directly suppresses neurotransmitter release from GABAergic terminals [30, 31]. To explore the involvement of CB1Rs on GABA^RVM^ neurons in EA-induced analgesia, we injected the rAAV-mDIx-cre-EYFP virus into the RVM of CB1R^flox/flox^ mice to selectively reduce CB1R expression on GABA^RVM^ neurons (Fig. 6A–D). In the CPP test (Fig. 6E–R), CFA- or CCI-induced CB1R^flox/flox^ control mice stayed in the matching chamber significantly longer after conditioned pairing with EA treatment (Fig. 6E–G, L–N, p < 0.05). However, knockdown of CB1Rs on GABA^RVM^ neurons antagonized EA-conditioned place preference (Fig. 6H–I, O–P, p > 0.05). Knockdown of CB1Rs on GABA^RVM^ neurons inhibited the analgesic effect of EA in CFA- and CCI-treated mice, as demonstrated in both the von Frey and hot plate tests (Fig. 6J–K, Q–R, p > 0.05). Knockdown of CB1Rs on GABA^RVM^ neurons did not influence locomotor or anxiety levels, as indicated by metrics such as average speed, total distance moved, time spent in the center, and center entries in the open field test (Fig. 6S–X, p > 0.05).

## Discussion

Pain is a universal health problem in the world, which has a great negative impact on the quality of life of patients [41]. EA, as an advanced treatment method based on traditional acupuncture, is widely used to relieve pain in clinical practice, and has the advantages of safety and wide applicability [42]. However, its specific analgesic mechanism is not completely clear, which limits its wider application and the formulation of optimal treatment scheme. Therefore, it is of great clinical significance to explore the analgesic mechanism of EA. This study is the first to show that EA induces analgesia by inhibiting GABA^RVM^ neurons. We found that electrical stimulation of the ST36 acupoint on the affected side significantly alleviated pain hypersensitivity and associated negative emotions caused by tissue inflammation and nerve injury. EA decreased c-Fos expression, neuronal calcium influx, and firing in GABAergic neurons in mouse models of inflammatory and neuropathic pain. Chemogenetic inhibition of GABA^RVM^ neurons reduces pain hypersensitivity under control and painful conditions. Chemogenetic activation of GABA^RVM^ neurons caused pain hypersensitivity in control mice and inhibited the analgesic effects of EA in both inflammatory and neuropathic pain models. These findings suggest that EA produces its analgesic effects by inhibiting GABA^RVM^ neurons. Our study also showed that knockout of CB1Rs on GABA^RVM^ neurons antagonized the analgesic and negative emotion relieving effects of EA. Thus, CB1R-mediated inhibition of GABA^RVM^ neurons likely contributes to the analgesic action of EA.

Pain is an unpleasant sensory and emotional experience associated with actual or potential tissue damage [43]. Many chronic pain conditions are associated with tissue inflammation (inflammatory pain) or damage to the somatic nervous system (neuropathic pain) [44]. EA therapy, an advanced approach to treating pain based on traditional acupuncture, is used alone or in conjunction with other treatments. EA therapy not only provides effective pain relief but also offers the benefits of versatility and safety compared to traditional drug treatments [45]. Various factors such as acupoints, stimulation frequency, and intensity of EA can influence the pain-relieving effects. EA on the ST36 acupoint can alleviate inflammatory pain through diverse mechanisms, while the parameters of EA stimulation are not uniform [37]. A recent study found that weak EA stimulation at the ST36 point with 0.5 mA/10 Hz effectively activated the vagal-adrenal axis, leading to the suppression of systemic inflammatory responses [38]. Our study demonstrated that weak EA stimulation at the ST36 point on the affected side, using 0.5 mA/10 Hz, significantly alleviated both CFA-induced inflammatory pain and CCI-induced neuropathic pain. This finding enhances the understanding of optimal acupoint selection and stimulation intensity for EA analgesic therapy.

The RVM is a key brain region for the endogenous nociceptive modulation, and GABA^RVM^ neurons are involved in downstream nociceptive control through inhibition of the off-cells [26]. GABA^RVM^ neurons facilitate mechanical pain by suppressing enkephalinergic/GABAergic interneurons in the dorsal horn [24]. We explored the role of GABA^RVM^ neurons in mediating EA's analgesic effects. Interestingly, we found that c-Fos expression and neuronal firing were increased in GABA^RVM^ neurons of mice with inflammatory and neuropathic pain. Also, the elevated calcium activity in GABA^RVM^ neurons was more pronounced in response to nociceptive stimuli. Activation of GABA^RVM^ neurons induced pain hypersensitivity in control mice. Conversely, inhibition of GABA^RVM^ neurons reduced pain hypersensitivity under physiological and painful conditions, further corroborating the involvement of GABA^RVM^ neurons in the facilitation of nociceptive transmission. EA treatment inhibited c-Fos expression, neuronal calcium activity, and neuronal firing of GABA^RVM^ neurons of mice in both pain models. Activation of GABA^RVM^ neurons diminished the analgesic effects of EA on both inflammatory and neuropathic pain. Therefore, EA likely produces its analgesic effects by inhibiting GABA^RVM^ neurons, offering a new mechanism for acupuncture analgesia.

Pain emotion belongs to the emotional dimension of pain, constituting the emotional and affective experiences induced by pain, including negative emotions such as disgust, fear, anxiety, and depression triggered by noxious or non-noxious stimuli [46]. Patients with chronic pain are three times more likely to experience negative emotions compared to those without pain [47]. Furthermore, negative emotions can exacerbate pain severity, and pain, in turn, intensifies negative emotions, creating a vicious cycle [48]. We found in this study that EA alleviated the negative emotions caused by pain, consistent with previous studies [49, 50]. Various neurotransmitters are involved in the development of pain emotions, which affect neuronal excitability through synaptic transmission, leading to abnormal functioning of the relevant neural circuits and acting as messengers for pain-induced negative emotional messaging [51]. Our study indicates that chemogenetic activation of GABA^RVM^ neurons diminishes the mood-enhancing effects of EA, suggesting that targeting GABA^RVM^ neurons could represent a novel mechanism by which EA alleviates pain-associated negative emotions.

CB1R is a prominently expressed G protein-coupled receptor in the brain [52] and is abundant in the RVM [53]. CB1Rs contribute to EA-induced analgesia across various brain regions, including the striatum, ACC, PAG, and primary somatosensory cortex (S1) [32]. The role of CB1Rs in the RVM during EA analgesia remains largely unexplored. The CB1R is predominantly located at presynaptic terminals and is preferentially expressed in GABAergic neurons [29]. Moreover, activation of CB1Rs in the RVM directly inhibits neurotransmitter release from GABA neuronal terminals [30, 31]. In the present study, we found that specific knockdown of CB1Rs on the GABA^RVM^ neurons antagonized the EA effects on analgesia and alleviation of negative emotions. Thus, EA may produce analgesic effects via inhibition of GABA^RVM^ neurons by activating CB1Rs.

## Conclusions

Our study indicates that GABAergic neurons in the RVM, especially CB1Rs on GABA^RVM^ neurons, significantly contribute to the analgesic effects of EA on inflammatory and neuropathic pain, suggesting a novel mechanism for EA-induced analgesia. This new information advances our mechanistic understanding of the EA analgesia and helps the design of new strategies to improve clinical management of pain.

## Supplementary Information

Additional file 1: Fig. 1. Ipsilateral (Ip) EA produces better analgesia effect than Opposite (Op) EA in mice with inflammatory and neuropathic pain. Fig. 2. Increased activity of GABA^RVM^ neurons in nociceptive hypersensitivity. Fig. 3. Inhibition of GABA^RVM^ neuron alleviates basal nociception. Fig. 4. Activation of GABA^RVM^ neurons induces hyperalgesia but not place avoidance behavior.

## Data Availability

The study's original data can be obtained from the corresponding authors upon reasonable request.

## Abbreviations

EA: Electroacupuncture
RVM: Rostral ventromedial medulla
CCI: Chronic constrictive injury
CB1R: Cannabinoid Type 1 Receptors
GABA^RVM^ neurons: GABAergic neurons within the RVM
NRM: Nucleus raphe magnus
NRGc: Reticular nucleus of the giant cell
CFA: Complete Freund's adjuvant
AAVs: Adeno-associated viruses
OD: Outer diameter
NA: Numerical aperture
CPP: Conditioned place preference
CPA: Conditioned place aversion
