# Supplementary material for: Electroacupuncture produces analgesic effects via cannabinoid CB1 receptor-mediated GABAergic neuronal inhibition in the rostral ventromedial medulla
Source: Chin Med. 2025 Mar 4;20:30. doi: 10.1186/s13020-025-01083-4 (PMC11881457; doi:10.1186/s13020-025-01083-4)
Supplement: Supplementary file 1 — Additional file 1: Fig. 1. Ipsilateral (Ip) EA produces better analgesia effect than Opposite (Op) EA in mice with inflammatory and neuropathic pain. Fig. 2. Increased activity of GABARVM neurons in nociceptive hypersensitivity. Fig. 3. Inhibition of GABARVM neuron alleviates basal nociception. Fig. 4. Activation of GABARVM neurons induces hyperalgesia but not place avoidance behavior. [file 13020_2025_1083_MOESM1_ESM.docx]

Supplementary Materials for

**Electroacupuncture Produces Analgesic Effects via Cannabinoid CB1 Receptor-Mediated GABAergic Neuronal Inhibition in the Rostral Ventromedial Medulla**

Kexing Wan et al.

* Corresponding author: Prof. Man Li

E-mail: liman73@mails.tjmu.edu.cn

Prof. Xianghong Jing

E-mail: jxhtjb@263.net

Prof. Hezhu

E-mail: zhuhe1983@hust.edu.cn

1. **Supplementary Methods**

**1.1** **Measurement of nociceptive thresholds**

Von Frey filaments were employed to measure mechanical allodynia, as previously reported ^[1]^. Briefly, the mice were kept in a clear glass frame (22 × 10 × 14 cm) for 30 minutes before the experiment. Six calibrated von Frey filaments were used in the up-and-down approach to assessing the mechanical withdrawal thresholds. The plantar of the hind paws were stimulated with von Frey fiber filaments perpendicularly. Positive responses included brisk paw withdrawal and paw licking.

The hot-plate test was used to assess the nociceptive heat threshold. Using a previously mentioned method, thermal withdrawal latency was measured ^[2]^. Each mouse was tested three times with a 10-minute interval.

**1.2 Open field test**

The open field arena was a square box (50 × 50 cm) with opaque Plexiglas walls. For each test, the mouse was placed in a corner of the box and allowed freely explore the surrounding environment. The mice's five-minute trajectories were recorded using Visu Track software, which analyzed how many times the mice entered the open central district and how long they stayed in the central district. After each test, the open field was cleaned with 75% ethanol to remove the remaining odor and avoid affecting the other mouse.

**1.3** **Conditioned place preference (CPP) test**

CPP procedure was conducted using the Ugo Basile CPP system and Video Mot software. Single Ugo Basile apparatus (external dimensions: 63 cm × 32 cm × 35 cm) consists of two compartments (internal dimensions 30 cm × 30 cm × 30 cm) differing by tactile and visual stimulation (floor structures and wall patterns) which are divided by the guillotine doors. One compartment has black and white horizontal striped walls and a floor with square 10 mm × 10 mm holes and the other has vertical stripe and a floor with strip 2 mm × 30 mm holes. Video Mot software enables live tracking of the animal and measures the time spent in each compartment, as well as the distance traveled.

Each mouse was first habituated to the chamber on days 1 and 2 (screening test) for 30 min each day. On day 3, each mouse was placed in the chamber and allowed to explore both chambers without stimulation (baseline) for 10 min. On days 4–6, for EA analgesia matching experiments, the mouse after EA treatment was confined to one chamber for 30 min. For the Chemogenetic experiment, each mouse was confined to the chamber with saline injection (i.p.) for 30 min in the morning, and 4 h later confined to another chamber with CNO injection (1.5 mg/kg, i.p.) for 30 min in the afternoon. On the final day (day 7), each mouse was allowed to explore both chambers for 10 min. Video Mot software enables live tracking of the animal and measures time spent in each compartment. CPP behaviors are presented as CPP scores defined by the formula: For EA analgesia matching experiments, CPP score = time in EA-paired chamber − time in NO-paired chamber in seconds. For the Chemogenetic study, CPP score = time in CNO-paired chamber − time in Saline-paired chamber in seconds.

**1.4** **Conditioned place aversion (CPA) test**

The equipment and software are the same as in CPP, and the procedure is similar to that of the CPP Chemogenetic experiment. Each mouse was first habituated to the chamber on days 1 and 2 (screening test) for 30 min each day. On day 3, each mouse was placed in the chamber and allowed to explore both chambers without stimulation (baseline) for 10 min. On days 4–6, each mouse was confined to the chamber with saline injection (i.p.) for 30 min in the morning, and 4 h later confined to another chamber with CNO injection (1.5 mg/kg, i.p.) for 30 min in the afternoon. On the final day (day 7), each mouse was allowed to explore both chambers for 10 min. VideoMot software enables live tracking of the animal and measures time spent in each compartment. CPA behaviors are presented as CPA scores defined by the formula: CPA score = time in CNO-paired chamber − time in Saline-paired chamber in seconds.

**1.5 Western blot analysis**

Western blotting was used to detect the levels of CB1R proteins. Protein lysates were extracted from the rostroventromedial Medulla (RVM) tissues. Protein samples were separated using a 10% gradient sodium dodecyl sulfate-polyacrylamide electrophoresis (SDS-PAGE) gel and transferred to a polyvinylidene difluoride (PVDF) membrane with a pore size of 0.45 m (Bio-Rad). The membranes were blocked in non-fat dry milk for 2 hours at 37°C before being treated with primary antibodies, including anti-CB1R (1:2000, 17978-1-AP, Proteintech), anti-actin (1:10,000, ab115777, Abcam) at 4°C overnight. Next, the membranes were incubated with Horseradish Peroxidase-conjugated secondary antibodies at room temperature for 1 hour and were visualized using Electro Chemo Luminescence detection reagent (34096, ThermoFisher Scientific, Waltham, MA, USA). Finally, Image Lab™ software 4.1 (Bio-Rad Laboratories, Inc., Hercules, CA, USA) was used to visualize protein bands. The density of protein bands was quantified using the Image J software.

**1.6** **Viral microinjection and optical fiber implantation**

To manipulate the activity of RVM neurons, adeno-associated viruses (AAVs) were injected into the RVM (AP, 5.72 mm; ML, ± 0.00 mm; DV, 5.70 mm) of mice, and behavioral tests were performed 3 weeks later. All injections were performed with a stereotaxic apparatus on mice under anesthesia produced by sodium pentobarbital (1% wt/vol), and body temperature was maintained with a heating pad. Ophthalmic ointment was applied to maintain eye lubrication. Viruses were injected at 30 nL/min using an air pressure system connected to a glass pipette (tip diameter 10–30 μm). After injection, the pipette was left in place for 10 min to allow diffusion. The mice were allowed to recover from anesthesia on a heating blanket before returning to the home cage.

For fiber photometry measurement, 100 nL of AAV2/9-hSyn-DIO-GCaMP6s-WPRE was injected into the RVM of Vgat-ires-Cre mice. After injection of AAV2/9-DIO-GCaMP6s, an optical fiber (outer diameter (OD) of 200 μm, numerical aperture (NA) of 0.37, Inper) was placed at the injection site.

For Chemogenetic experiments, AAV2/9-hSyn-DIO-hM3Dq-mCherry, AAV2/9-hSyn-DIO-hM4Di-mCherry or AAV2/9-hSyn-DIO-mCherry was injected into the RVM of Vgat-ires-Cre mice. These viruses were obtained from Brain Case Biotechnology Co. Ltd (Wuhan, China). Clozapine N-oxide (CNO, Enzo Life Sciences, Inc.) was dissolved in saline to a concentration of 0.3 mg/mL. For activating or inhibiting GABA^RVM^ neurons, DREADD activation was achieved via intraperitoneal injection of CNO in Vgat-ires-Cre mice at 3 mg/kg, and the withdrawal thresholds were measured 1 h before and 1 h after each CNO injection.

**1.7** **Fiber photometry *in vivo* calcium imaging**

Two weeks after the AAV-hSyn-DIO-GCaMP6s-WPRE virus was injected into the RVM of Vgat-ires-Cre mice, an optical fiber (200 μm OD, 0.37 NA) was implanted into the RVM. Each mouse was allowed to recover for 1 week before recording. A fiber photometry system was used for recording. Ca^2+^ transient signal was recorded simultaneously after the hindpaw was stimulated with Von Frey or pinch. For noxious mechanical stimulation (pinch), an alligator clip (Generic Micro Steel Toothless Alligator Test Clips 5AMP, Amazon) producing 340 g force was applied to the ventral skin surface between the footpad and the heel ^[3]^. At the end of the experiment, all animals were perfused. Only data from animals with correct optical fiber implantation sites and virus expression were included in the analysis. The change in Ca^2+^ transient value (ΔF/F) from 4 s preceding the onset of hindpaw withdrawal to 8 s after the onset of pinch stimulation was derived by calculating (F−F0)/F0 for each pinch stimulation, where F0 is the median Ca^2+^ transient at 4 s preceding pinch stimulation to its onset. The ΔF/F values of all pinch stimuli were then averaged and plotted with a shaded area indicating the SEM.

**1.8** ***In vivo* electrophysiological recordings**

Broadband neural signals (0.3 Hz–7.5 kHz) were recorded simultaneously from implanted 16-channel arrays using a data-acquisition system (Zeus, Bio-Signal Technologies: McKinney, TX, U.S.A.) at a sampling rate of 30 kHz. Spike isolation was achieved with a high-pass filter set at 300 Hz, and real-time spike sorting was conducted using principal component analysis (PCA). Subsequent spike sorting refinement was performed with Offline Sorter (Plexon: Dallas, TX, U.S.A.). Data analysis was carried out in NeuroExplorer 5 (Nex Technologies: Boston, MA, U.S.A.). The specific brain regions targeted for electrode implantation was the RVM (AP, 5.72 mm; ML, ± 0.00 mm; DV, 5.70 mm). The electrodes, constructed with 16 individually insulated nichrome wires (35 μm inner diameter) with impedance ranging from 300 to 900 Kohm (Stablohm 675, California Fine Wire, U.S.A.), were arranged in arrays of 16 micro-wires in a 3 × 5 × 5 × 3 pattern with approximately 200 μm spacing between wires. These wires were connected to an 18-pin connector (Mil-Max) and secured in place with dental cement. Subject mice were placed in a cylindrical box lined with copper mesh, allowing them to move freely without disturbance when multichannel electrical signals were recorded.

1. **Supplementary Figures**


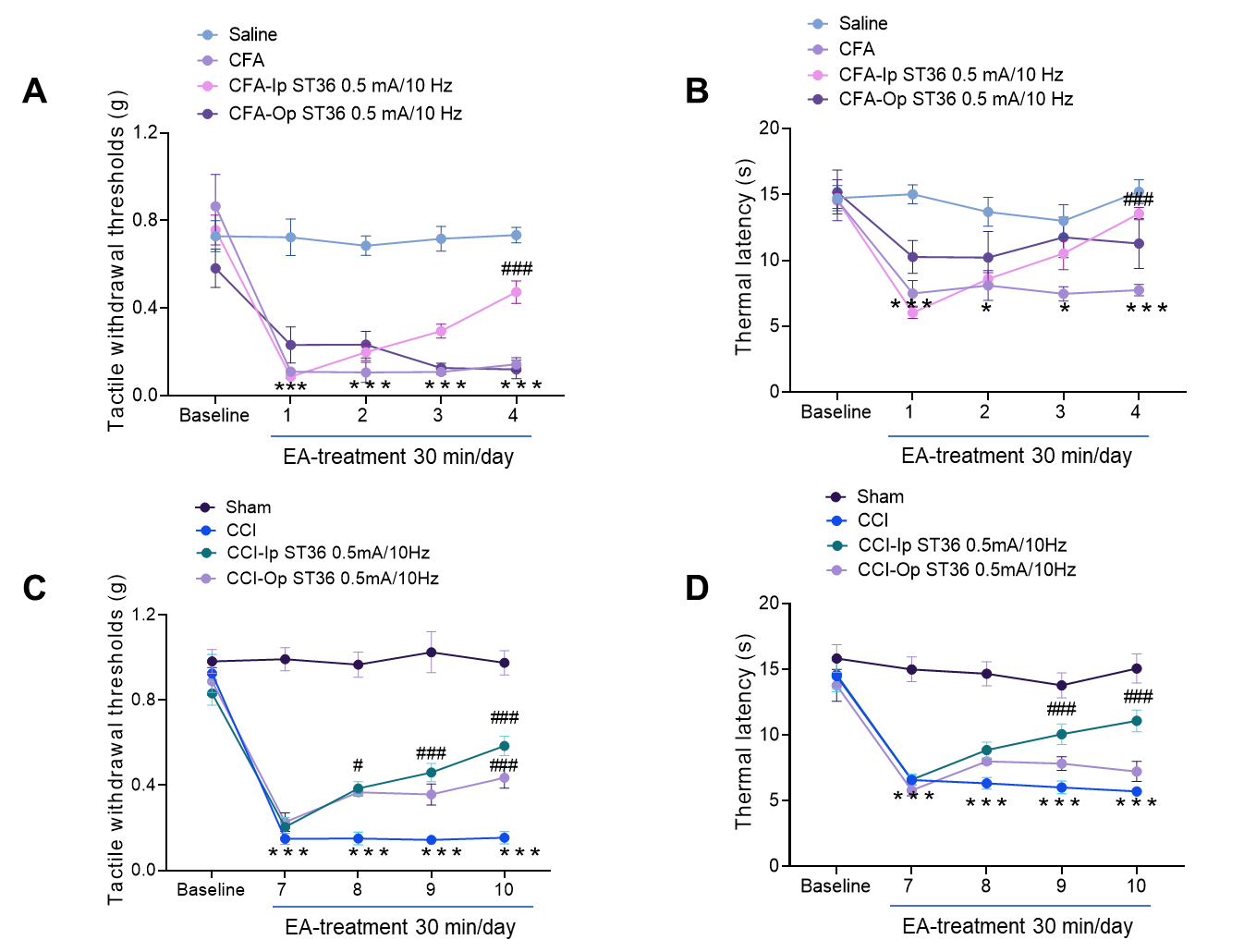


**Supplementary Fig. 1. Ipsilateral (Ip) EA produces better analgesia effect than Opposite (Op) EA in mice with inflammatory and neuropathic pain.** (A) Time course of changes in the tactile withdrawal thresholds based on von Frey tests in the CFA model. (B) Time course of changes in the thermal withdrawal latency based on hot plate test in CFA model. (C) Time course of changes in the tactile withdrawal thresholds based on von Frey tests in the CCI model. (D) Time course of changes in the thermal withdrawal latency based on hot plate test in CCI model. *** *p* < 0.001, * *p* < 0.05 vs. Saline or Sham control group, # *p* < 0.05, ### *p* < 0.001 vs. CFA or CCI group, revealed by two-way ANOVA with Bonferroni’s post hoc test, n = 6 mice per group.


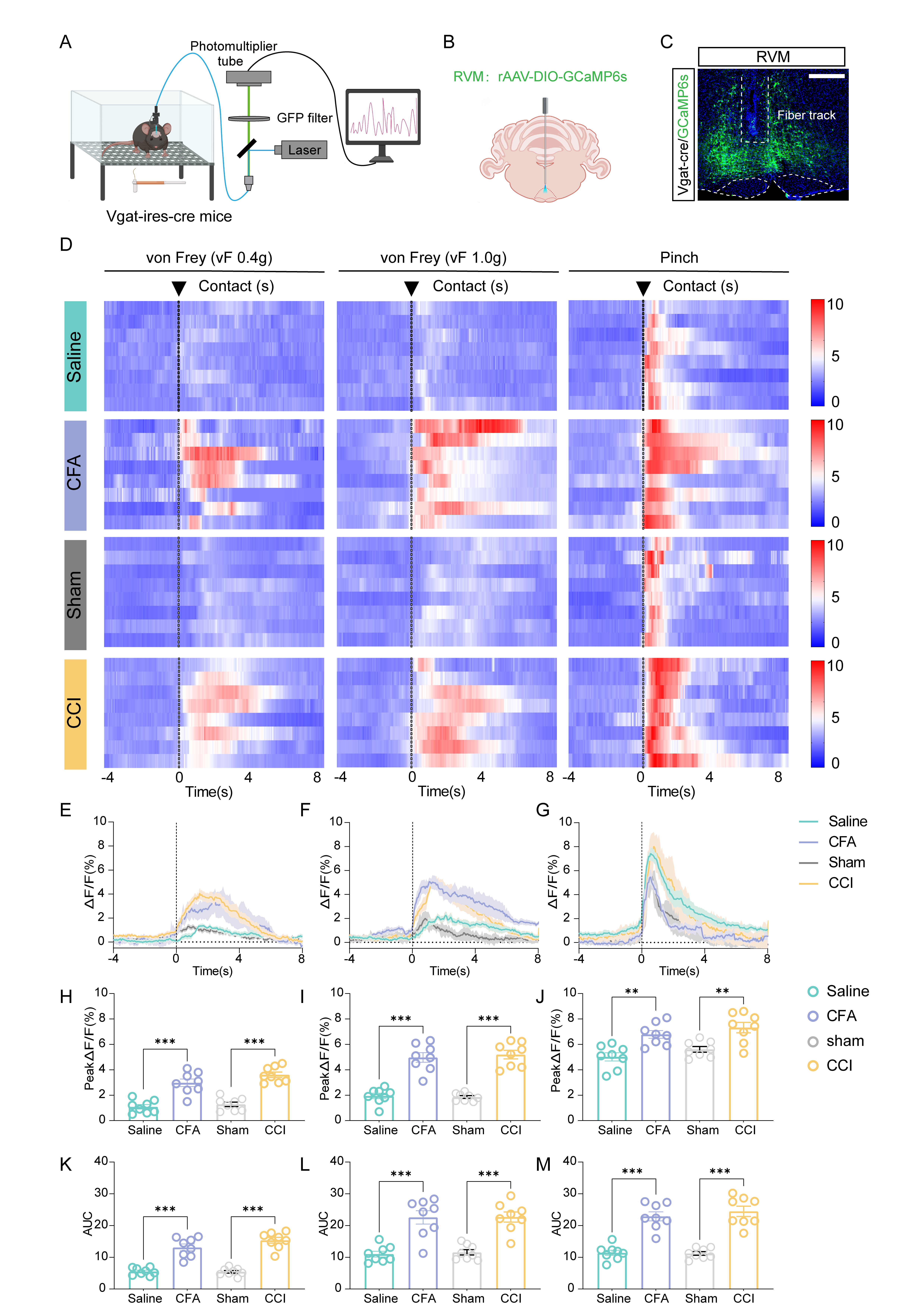


**Supplementary Fig. 2. Increased activity of** **GABA^RVM^ neurons in pain hypersensitivity**

**(A)** The diagram outlining the fiber photometry configuration. Calcium (Ca^2+^) transients were measured in vgat-ires-cre mice's RVM neurons that express GCaMP6s. **(B)** The illustration of the fiber photometry location within the RVM. **(C)** The representative images depicting the injection site within the RVM using AAV-DIO–GCaMP6s, Scale bars = 500 µm. **(D)** The average activity of GABAergic neurons in response to specific stimuli was analyzed. Calcium signaling events were recorded and synchronized with the moment of hindpaw stimulation using a pinch, von Frey (0.4 g), or von Frey (1.0 g) (represented by black arrowheads). A total of 8 trials from 4 mice were collected and organized according to the time of peak neural activity. The arrowheads point to the beginning of the stimulus. **(E-G)** Representative fluorescence signals (ΔF/F) of GCaMP6s recorded from GABA^RVM^ neurons aligned to von Frey 0.4 g (E), von Frey 1.0 g (F), and pinch (G). Number of trials recorded is the same as in (D). **(H-J)** Averaged peak ΔF/F per second of GCaMP6s fluorescence signals from GABA^RVM^ neurons aligned to von Frey 0.4 g (H), von Frey 1.0 g (I), and pinch (J). (**K-M)** The area under the curve (AUC) per second of GCaMP6s fluorescence signals from GABA^RVM^ neurons aligned to von Frey 0.4 g (K), von Frey 1.0 g (L), and pinch (M). ** *p* < 0.01 and *** *p* < 0.001 vs. Saline or Sham control group, two-tailed unpaired t-test, n = 8. All data are shown as mean ± SEM.


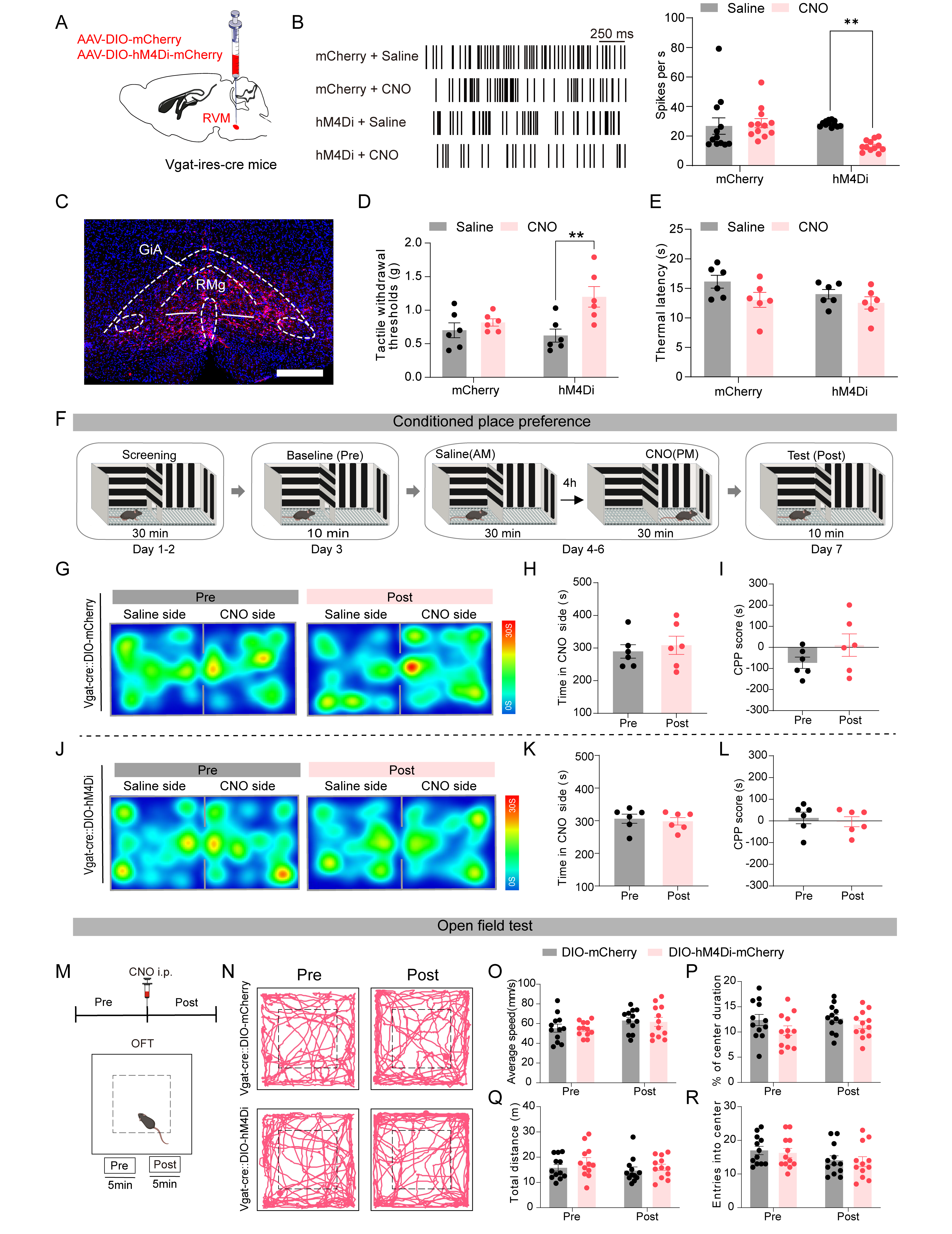


**Supplementary Fig. 3. Inhibition of** **GABA^RVM^ neuron alleviates basal nociception**

**(A)** The diagram illustrating the stereotaxic injection of AAV-DIO-hM4Di-mCherry or AAV-DIO-mCherry viruses into the RVM region of vgat-ires-cre mice. **(B)** Representative recording of spontaneous spikes and data showing GABA^RVM^ neuronal firing rates in the vgat-cre::DIO-mCherry and vgat-cre::DIO-hM4Di mice treated with Saline or CNO (n = 12 cells from 3 mice for RVM:: mCherry+Saline; n = 12 cells from 3 mice for RVM:: mCherry+CNO; n = 12 cells from 3 mice for RVM:: hM4Di+Saline; n = 12 cells from 4 mice for RVM:: hM4Di+CNO). Scale bars = 250 ms. **(C)** The representative fluorescence image shows the injection site of the virus. Scale bars = 500 μm. **(D-E)** The effect of chemogenetic inhibition of GABA^RVM^ neurons on the tactile withdrawal thresholds (D) and the thermal withdrawal latency (E), n = 6 mice per group. For B, D-E, ***p* < 0.01 vs. Saline-treatment within mCherry or hM4Di group, two-way ANOVA followed by Bonferroni’s *post hoc* tests. **(F)** The schematic diagram of the experiment design for the conditioned place preference (CPP) test. **(G)** The representative tracking maps of vgat-cre::DIO-mCherry mice in the CPP before and after Saline- and CNO-conditioning. **(H-I)** The time spent in the CNO-conditioned chamber (H) and CPP score (I) of vgat-cre::DIO-mCherry mice. **(J)** The representative tracking maps of vgat-cre::DIO-hM4Di mice in the CPP before and after Saline- and CNO-conditioning. **(K-L)** The time spent in the CNO-conditioned chamber (K) and CPP score (L) of vgat-cre::DIO-hM4Di mice. n = 6. **(M)** The schematic diagram of the experiment design for the open field test (OFT). **(N)** The representative tracking maps of vgat-cre::DIO-mCherry and vgat-cre::DIO-hM4Di mice in the OFT before and after CNO injection. **(O-R)** The effect of chemogenetic inhibition of GABA^RVM^ neuron on the average speed (O), the ratio of time spent in the center (P), the total distance moved (Q), and entries into the center (R) in the open field, n = 12 mice per group. All data are shown as mean ± SEM.


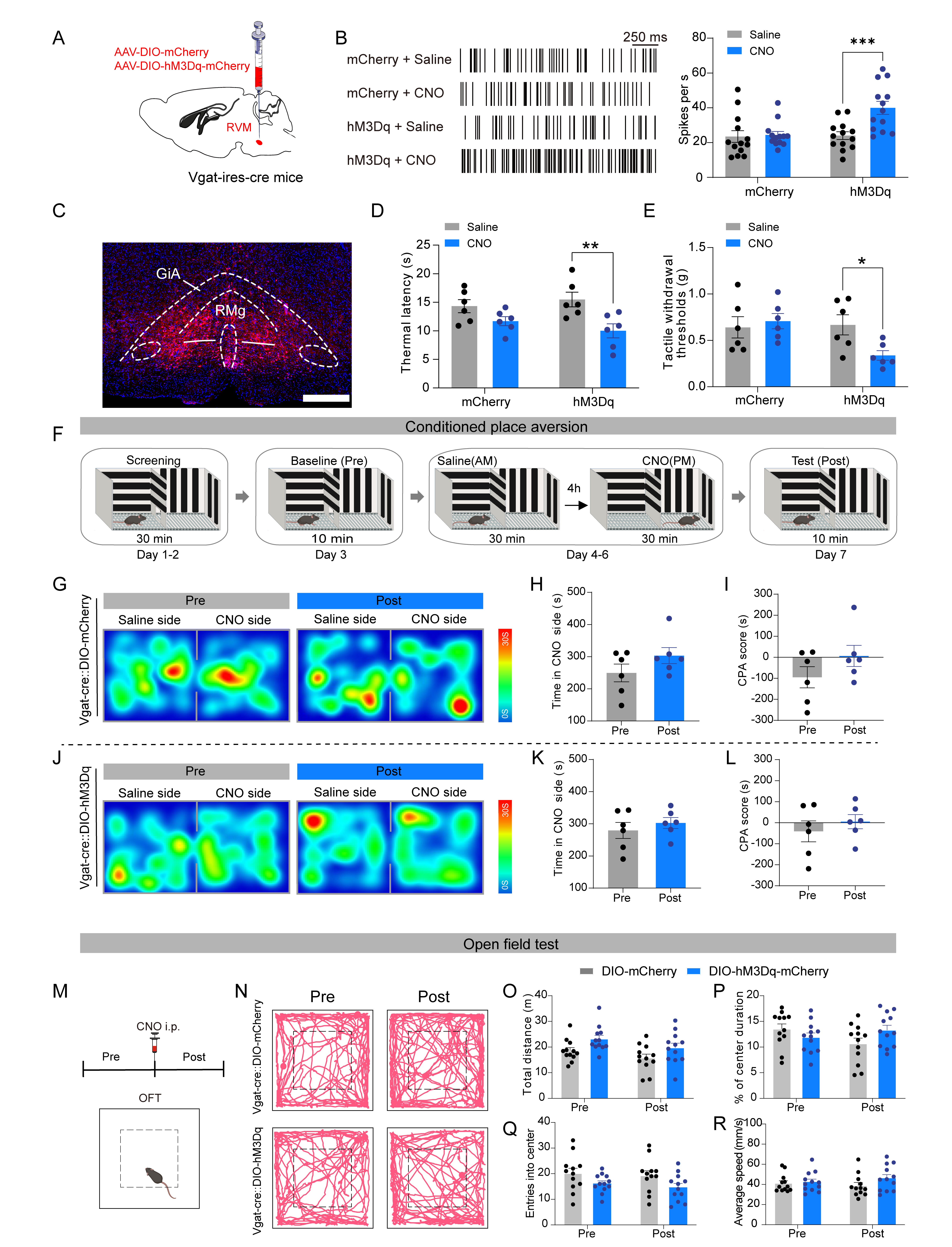


**Supplementary Fig. 4. Activation of** **GABA^RVM^ neurons induces hyperalgesia but not place** **avoidance behavior**

**(A)** The diagram illustrating the stereotaxic injection of AAV-DIO-hM3Dq-mCherry or AAV-DIO-mCherry viruses into the RVM region of vgat-ires-cre mice. **(B)** Representative recording of spontaneous spikes and data showing GABA^RVM^ neuronal firing rates in the vgat-cre::DIO-mCherry and vgat-cre::DIO-hM3Dq mice treated with Saline or CNO (n = 13 cells from 3 mice for RVM:: mCherry+Saline; n = 13 cells from 3 mice for RVM:: mCherry+CNO; n = 13 cells from 3 mice for RVM:: hM3Dq+Saline; n = 13 cells from 3 mice for RVM:: hM3Dq+CNO). Scale bars = 250 ms. **(C)** The representative fluorescence image shows the injection site of the virus. Scale bars = 500 μm. **(D-E)** The effect of chemogenetic activation of GABA^RVM^ neurons on the tactile withdrawal thresholds (D) and the thermal withdrawal latency (E), n = 6. For B, D-E, **p* < 0.05, ***p* < 0.01, and ****p* < 0.001 vs. saline treatment within mCherry or hM3Dq group, two-way ANOVA followed by Bonferroni’s *post hoc* tests. **(F)** The schematic diagram of the experiment design for the conditioned place avoidance (CPA) test. **(G)** The representative tracking maps of vgat-cre::DIO-mCherry mice in the CPP before and after Saline- and CNO-conditioning. **(H-I)** The time spent in the CNO-conditioned chamber (H) and CPA score (I) of vgat-cre::DIO-mCherry mice. **(J)** The representative tracking maps of vgat-cre::DIO-hM3Dq mice in the CPA before and after Saline- and CNO-conditioning. **(K-L)** The time spent in the CNO-conditioned chamber (K) and CPA score (L) of vgat-cre::DIO-hM3Dq mice. n = 6. **(M)** The schematic diagram of the experiment design for the open field test (OFT). **(N)** The representative tracking maps of vgat-cre::DIO-mCherry and vgat-cre::DIO-hM3Dq mice in the OFT before and after CNO injection. **(O-R)** The effect of chemogenetic activation of GABA^RVM^ neuron on the total distance moved (O), the ratio of time spent in the center (P), the entries into the center (Q), and the average speed (R) in the open field, n = 12 mice per group. All data are shown as mean ± SEM.

**Reference**

[1] Bennett GJ, Xie YK. A peripheral mononeuropathy in rat that produces disorders of pain sensation like those seen in man [J]. Pain, 1988, 33(1): 87-107.

[2] Chaplan SR, Bach FW, Pogrel JW, et al. Quantitative assessment of tactile allodynia in the rat paw [J]. J Neurosci Methods, 1994, 53(1): 55-63.

[3] Huang T, Lin SH, Malewicz NM, et al. Identifying the pathways required for coping behaviours associated with sustained pain [J]. Nature, 2019, 565(7737): 86-90.
